# In Situ Construction of Nitrogen-Doped and Zinc-Confined Microporous Carbon Enabling Efficient Na^+^-Storage Abilities

**DOI:** 10.3390/ijms24108777

**Published:** 2023-05-15

**Authors:** Wan-Ling Liao, Mohamed M. Abdelaal, Rene-Mary Amirtha, Chia-Chen Fang, Chun-Chen Yang, Tai-Feng Hung

**Affiliations:** 1 Battery Research Center of Green Energy, Ming Chi University of Technology, 84 Gungjuan Rd., New Taipei City 24301, Taiwan; 2 Tabbin Institute for Metallurgical Studies (TIMS), Tabbin, Helwan 109, Cairo 11421, Egypt; 3 Material and Chemical Research Laboratories, Industrial Technology Research Institute, 195, Sec. 4, Chung Hsing Rd., Hsinchu 31040, Taiwan; 4 Department of Chemical Engineering, Ming Chi University of Technology, 84 Gungjuan Rd., New Taipei City 24301, Taiwan; 5 Department of Chemical and Materials Engineering, Chang Gung University, 259 Wenhua 1st Rd., Taoyuan 33302, Taiwan

**Keywords:** sodium-ion batteries, nitrogen-doped porous carbon, thermal pyrolysis, zeolitic imidazolate framework-8, clean energy technology

## Abstract

Benefiting from the additional active sites for sodium-ion (Na^+^) adsorption and porous architecture for electrolyte accessibility, nitrogen-doped porous carbon has been considered the alternative anode material for Na^+^-storage applications. In this study, nitrogen-doped and zinc-confined microporous carbon (*N,Z*-MPC) powders are successfully prepared by thermally pyrolyzing the polyhedral ZIF-8 nanoparticles under an argon atmosphere. Following the electrochemical measurements, the *N,Z*-MPC not only delivers good reversible capacity (423 mAh/g at 0.02 A/g) and comparable rate capability (104 mAh/g at 1.0 A/g) but also achieves a remarkable cyclability (capacity retention: 96.6% after 3000 cycles at 1.0 A/g). Those can be attributed to its intrinsic characteristics: (a) 67% of the disordered structure, (b) 0.38 nm of interplanar distance, (c) a great proportion of sp^2^-type carbon, (d) abundant microporosity, (e) 16.1% of nitrogen doping, and (f) existence of sodiophilic Zn species, synergistically enhancing the electrochemical performances. Accordingly, the findings observed here support the *N,Z*-MPC to be a potential anode material enabling exceptional Na^+^-storage abilities.

## 1. Introduction

To achieve net-zero emissions globally by 2050, employing renewable energy to generate clean electricity is regarded as a straightforward approach [1,2,3]. With the incessant development of renewable energy sources, highly stable and cost-effective energy storage devices (ESDs) are urgently desired for smoothing the intermittent electricity output, providing frequency regulation services and improving system reliability [4]. According to the information disclosed in the global energy storage database, it is seen that 198 operational projects utilized lithium- or sodium-ion batteries as the alternative ESDs. Especially, 95% of operational projects are based on lithium-ion batteries (LIBs) [5]. It can be ascribed to their fast response time, no geographical restrictions, and adjustable output based on the requirements as compared to other technologies [6].

Except for energy storage applications, they also serve as electricity resources for electric vehicles (EVs). Due to the rapid growth of the LIBs market, it is noticed that the price of lithium carbonate, a critical chemical for lithium-ion batteries, greatly climbed to around CNY 600,000 per ton in November of 2022. Although it declined to CNY 365,500 in early March of 2023, such a price is still about five times higher than that in March of 2021 [7]. Apart from the LIBs, the successful manufacturing of sodium-ion batteries (SIBs) is recently announced [8]. Being an alternative charge carrier to lithium, the benefits of sodium can be attributed to the material abundance, low cost, and electrochemical similarities [9]. Despite the growing progress in the SIBs, the implementation of this technology still remains challenging due to the kinetics mismatch between the anode and cathode.

Carbonaceous materials have been regarded as the most promising anodes for SIBs because of their diverse structures and properties, as well as extensive availability and low cost [10,11]. Among them, hard carbon (HC) was first demonstrated as a feasible intercalation anode for SIBs in 2000 [12]. Benefiting from the enriched microcrystalline structures (i.e., providing more defects and active sites to store sodium ions) and low operating potential (~0.1 V vs. Na/Na^+^), a reversible capacity of 300 mAh/g was achieved, which is close to that for lithium insertion in graphitic carbon anode materials [12]. Nonetheless, the diffusion of Na^+^ within the HC is quite sluggish, causing poor rate capability. Besides that, the large stress generated by Na^+^ insertion/extraction will also result in rapid structural collapse and thus leads to poor cycling stability [13,14]. To address those concerns, nitrogen (N)-doped carbon materials have attracted intense attention [15,16,17,18,19]. It is well-known that the configurations of N presented in graphitic carbon included pyrrolic N (N-5), quaternary N (N-Q), and pyridinic N (N-6), which can be found at defects, edge sites, and skeletons in graphite planes, respectively [20]. So far, it is always believed that N-6 plays a major role in the storage of alkali metal ions [21,22]. However, even better performance can be achieved when the content of N-5 is high enough, which is attributed to the introduction of N-5 that can enhance the conductivity, enlarge the interlayer distance, and increase defects for sodium storage [20].

ZIF-8, one of the zeolitic imidazolate frameworks with a sodalite zeolite-like structure, is constructed by Zn ions (Zn^2+^) and imidazolate (C_3_H_3_N_2_^−^) linkers. Given a variety of morphologies, three-dimensional (3D) frameworks, and high microporosities, it is widely functioned as the starting material for efficiently preparing the N-doped porous carbon (*N*-PC) by thermal pyrolysis under an inert atmosphere [23,24,25,26]. Owing to the good thermal stability and self-templating behavior, the morphology and textural features of the resulting *N*-PC were not significantly altered [27]. Furthermore, the amount of N present in the *N*-PC is much greater than the one prepared using urea as the N source, which is due to the high tetrahedrally coordinated structures among the Zn^2+^ and C_3_H_3_N_2_^−^ [28].

Inspired by the concepts (i.e., porous characteristics and highly N-doped amounts) mentioned above, the polyhedral ZIF-8 nanoparticles were first synthesized in the present study under ambient conditions. Unlike the organic solvent (e.g., methanol, ethanol, dimethylformamide, etc.) generally utilized for synthesizing the ZIF-8, deionized (DI) water was selected here as a green medium because of the cost and environmental considerations [29,30,31]. Subsequently, the N-doped and Zn-confined microporous carbon (*N,Z*-MPC) was prepared through thermal pyrolysis of ZIF-8 under an argon atmosphere. After systemically analyzing the crystallinity, morphological, and physicochemical properties of *N,Z*-MPC, the possible reasons affecting the electrochemical performances and storage mechanism were discussed. On the basis of the results reported in this study, it is rationally believed that the *N,Z*-MPC can be used as an alternative anode material for sodium storage applications.

## 2. Results and Discussion

### 2.1. Characterizations of Zeolitic Imidazolate Framework-8 (ZIF-8)

**Figure 1:**
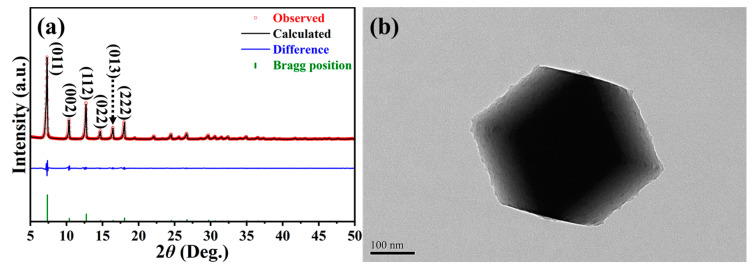
(**a**) Refined PXRD patterns and (**b**) low-magnification TEM micrograph of ZIF-8. Scale bar of (**b**) is 100 nm.

To confirm the successful synthesis of ZIF-8 within an aqueous medium, a variety of characterizations, including crystallography, morphology, textural property, and chemical environment, were systemically conducted. First of all, Figure 1a illustrates the refined PXRD pattern that was recorded from 2*θ* between 5° and 50° to identify the crystallinity and purity. As can be seen, the observed pattern matched well with the simulated one, which was retrieved from the crystallography open database (COD ID: 7111968) [32]. In addition, the relatively low values in terms of goodness of fit (GOF: 2.48) and reliability factors (*R*_exp_: 2.90%, *R*_wp_: 7.20%, and *R*_p_: 5.53%) were also obtained. The predominant peaks located around the 2*θ* of 7.3°, 10.4°, 12.7°, 14.7°, 16.4°, and 18.1° corresponded to the (011), (002), (112), (022), (013), and (222) crystallographic planes of ZIF-8, respectively [33]. Besides that, it is noticed that the undesired signals were not detected, indicating that high-quality ZIF-8 was successfully synthesized using an aqueous medium, even under ambient conditions. The micrographs captured from SEM and TEM are displayed in Figure S1a and Figure 1b, respectively. As revealed, the synthesized ZIF-8 appeared as a polyhedral architecture with well-defined facets and sharp edges (see Figure S1a). Moreover, their particle sizes were estimated to be 300 nm to 500 nm, which was similar to the range reflected in Figure 1b. The nitrogen adsorption–desorption measurement was carried out to accurately determine the specific surface area (SSA) and pore-size distribution, in which the corresponding isotherm is plotted in Figure S1b. Following the IUPAC classification, the isotherm can be categorized as Type I. On the other hand, the SSA value received from the Brunauer-Emmett-Teller (BET) method was found to be 1484 m^2^/g, while the pore-size distribution analyzed by a *t-plot* method revealed a range between 0.3 nm and 0.9 nm (see the inset of Figure S1b). Those results suggested that the ZIF-8 synthesized in the present study possessed the microporous feature, which was consistent with the others prepared through organic solvents [30,33].

**Figure 2:**
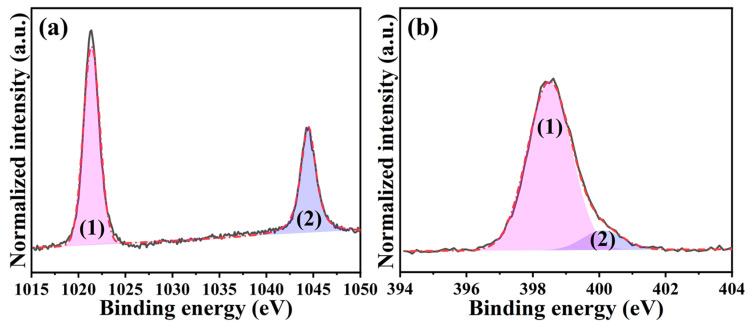
High-resolution XPS spectra of ZIF-8: (**a**) Zn 2p ((1) for Zn 2p_3/2_ and (2) for Zn 2p_1/2_) and (**b**) N 1s ((1) for N-Zn coordination and (2) for non-deprotonated -NH bonding).

The chemical composition and environment of synthesized ZIF-8 were analyzed using X-ray photoelectron spectroscopy (XPS). The full-range XPS spectrum in Figure S2 indicates the presence of Zn, O, N, and C, where the atomic percent of each element was 5.8%, 8.6%, 22.0%, and 63.6% in turn. Accordingly, the calculated N/Zn ratio is 3.8, approaching the theoretical value in ZIF-8 [34]. Again, the successful synthesis of ZIF-8 is verified. Figure 2a,b further illustrates the high-resolution Zn 2p and N 1s spectra, respectively. The former showed two distinct peaks in the binding energies of 1021.4 eV and 1044.5 eV, which were assigned to (1) Zn 2p_3/2_ and (2) Zn 2p_1/2_. The difference between these peaks was calculated to be 23.1 eV, meaning +2 of valence state for the Zn [35]. This can be attributed to the fact that the metal Zn center is Zn^2+^ in the ZIF-8 [36]. As for the latter, two deconvoluted peaks fitted by the Gaussian-Lorentzian method were centered at 398.5 eV and 400.1 eV, which were associated with (1) N-Zn coordination and (2) non-deprotonated -NH- bonding, respectively [34,37]. Considering the integrated area ratio among those peaks, it is found that 90.5% of N existed in N-Zn coordination, verifying that the majority of N was coordinated with Zn^2+^ to build ZIF-8.

### 2.2. Characterizations of Nitrogen-Doped and Zinc-Confined Microporous Carbon (N,Z-MPC)

**Figure 3:**
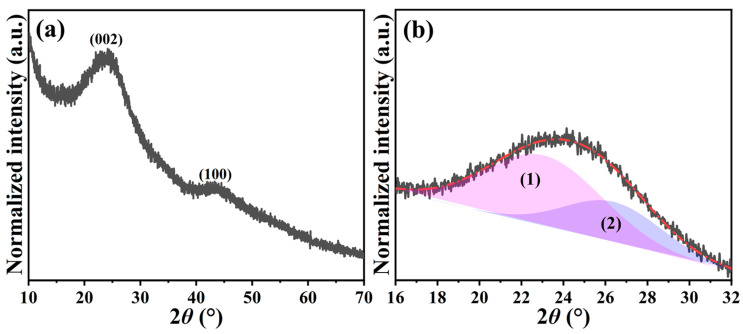
(**a**) Normalized PXRD pattern of *N,Z*-MPC and (**b**) its magnified (002) peak analyzed by Gaussian-Lorentzian method, where (1) and (2) stand for disordered and graphitic regions, respectively.

The *N,Z*-MPC powders were directly acquired through thermal pyrolysis of ZIF-8 under an argon atmosphere; consequently, their characteristics, such as crystallography, morphology, textural property, and chemical environment, were also carefully scrutinized. Figure 3a depicts the normalized PXRD pattern, which was collected in 2*θ* range of 10° and 70°. As revealed, only two broad peaks representing the (002) and (100) crystallographic planes of carbon (JCPDS No.: 41-1487) were detected [38]. When analyzing the (002) peak using the Gaussian-Lorentzian method from 2*θ* of 16° to 32°, it can be seen that the 2*θ* value was fitted well to be 24.18° (see Figure S3), corresponding to the *d*-spacing of 0.37 nm. In order to clearly classify the crystal region within the *N,Z*-MPC, two deconvoluted peaks correlated to disordered (1) and graphitic (2) regions were discriminated, as shown in Figure 3b. The obtained results are also summarized in Table S1. As expected, the higher area proportion of disordered regions (i.e., 67%) was reflected. Moreover, another effective approach to evaluate the disorder degree of carbonaceous materials by using empirical parameter *R* was proposed. On the basis of the definition, the *R*-value is received from the ratio of the height of the (002) diffraction peak to the background [39,40]. The low *R-*value means that the parallel-stacked graphene sheet structures that existed within the carbonaceous materials were less. Accordingly, the lower the *R*-value, the more the disordered structure will be contained. By calculating, the *R*-value of *N,Z*-MPC was 2.96, which was significantly lower than the mesophase pitch-derived carbon (i.e., 5.47) prepared under the same pyrolysis temperature [40].

**Figure 4:**
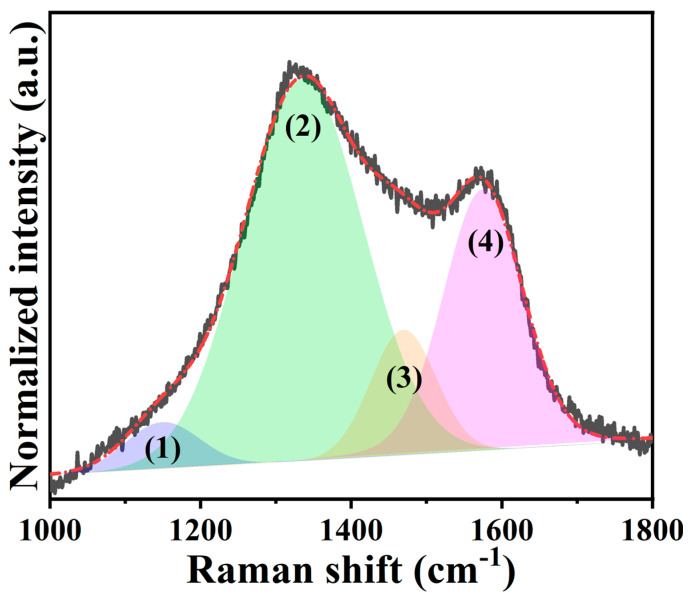
Raman spectrum of *N,Z*-MPC fitted by Gaussian-Lorentzian method.

Raman identification is a general strategy that can straightforwardly understand the graphitization degree of carbonaceous materials. The normalized Raman spectrum plotted in Figure 4 reveals two obvious peaks that appeared at 1336 cm^−1^ and 1575 cm^−1^, which were the typical signals for the D and G bands. Considering the intensity ratio among these peaks (i.e., *I*_D_/*I*_G_), it is found that the value was calculated to be 1.32. This result suggests that less graphitization degree of *N,Z*-MPC shows a good agreement with the evidence given in Figure 3b. Utilizing carbonaceous material as the active species of the electrode, the graphitization degree is well-recognized as one of the significant factors affecting its electronic conductivity. Consequently, it is imperative to discuss the proportion of sp^3^ to sp^2^ within the *N,Z*-MPC, even though the aforementioned results already showed its highly disordered structure. To figure out this idea, the Raman spectrum analyzed by the Gaussian-Lorentzian method was further deconvoluted into four peaks, which were labeled as peaks (1) to (4) in Figure 4. Among them, peaks (1) and (3) are associated with sp^3^-type carbon, while the others are related to sp^2^-type carbon [38,41]. The integrated area ratio of sp^3^ to sp^2^ (*A*_sp_^3^/*A*_sp_^2^) has been demonstrated to provide insightful information on the nature of carbonaceous materials, e.g., a low *A*_sp_^3^/*A*_sp_^2^ ratio gives that a larger amount of carbon exists as sp^2^-type [42]. As a result, the calculated *A*_sp_^3^/*A*_sp_^2^ ratio was about 0.17, signifying that the *N,Z*-MPC still retained a great proportion of sp^2^-type carbon, even intrinsically with less crystallinity.

**Figure 5:**
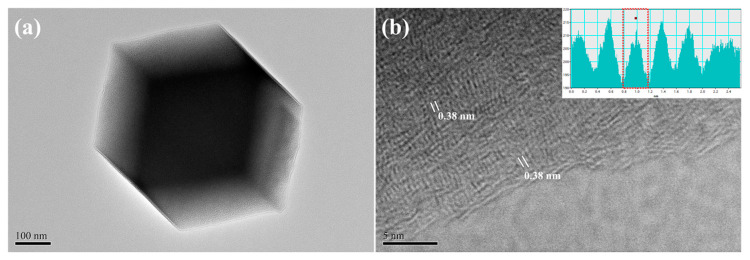
(**a**) Low-magnification and (**b**) high-resolution TEM micrographs of *N,Z*-MPC. Scale bar: (**a**) 100 nm and (**b**) 5 nm.

The morphological observations of *N,Z*-MPC were performed through SEM-EDX and TEM. In comparison with the original ZIF-8, it can be seen that not only the polyhedral architecture but also the size observed from the *N,Z*-MPC (see Figure S4a and Figure 5a) were not significantly varied even after 800 °C of pyrolysis temperature. Moreover, the elements of C, O, Zn, and N were well-distributed within *N,Z*-MPC, as shown in Figure S5, suggesting that the Zn was confined inside the microporous carbon. To facilitate sodium-ion intercalation and de-intercalation, it is reported that the *d*-spacing of carbonaceous materials should be more than 0.37 nm [43]. With further magnifying, about 0.38 nm of the distance between adjacent lattices was revealed, as pointed out in Figure 5b, indicating that our *N,Z*-MPC meet the basic criteria towards sodium-ion transportations. No matter the carbonaceous materials were synthesized by which kinds of precursors, it has been proven that the textural properties, including the SSA value, pore size, and distribution function, are critical factors in influencing the initial Coulombic efficiency, ionic mobility, reversible capacity, etc. [14,44,45]. Figure S4b shows the nitrogen adsorption–desorption isotherm (Type I [46]). It is noticed that the SSA value dramatically declined from 1484 m^2^/g to 441 m^2^/g, representing a similar tendency in ZIF-8-derived carbon materials [47]. Nevertheless, a similar pore-size distribution was detected from *N,Z*-MPC (i.e., 0.3 nm ~ 0.9 nm, see the inset of Figure S4b), solidly supporting a microporous characteristic that was still reserved.

**Figure 6:**
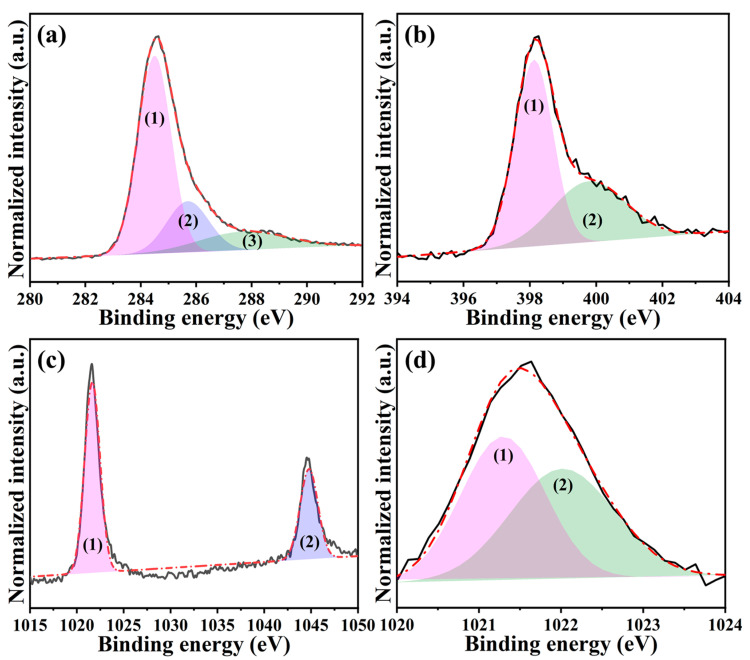
High-resolution XPS spectra of *N,Z*-MPC: (**a**) C 1s ((1) for C-C, (2) for C-N, and (3) for C-O/ C=O), (**b**) N 1s ((1) for pyridinic-N and (2) for pyrrolic-N), (**c**) Zn 2p ((1) for Zn 2p_3/2_ and (2) for Zn 2p_1/2_), and (**d**) magnified Zn 2p_3/2_ peak ((1) for Zn^0^ and (2) for ZnO).

To explore the chemical composition and environment of *N,Z*-MPC, the full-range XPS survey and high-resolution analysis were conducted. The former (Figure S6) shows the existence of C, N, O, and Zn with atomic percent of 74.2%, 16.1%, 7.4%, and 2.2%, respectively. As for the latter, the C 1s spectrum in Figure 6a was fitted into three peaks at 284.5 eV, 285.7 eV, and 287.7 eV, which was attributed to C-C (1), C-N (2), and C-O/C=O (3) [37]. Secondly, two deconvoluted peaks associated with pyridinic-N (1) and pyrrolic-N (2) species were found at 398.1 eV and 399.8 eV from the N 1s spectrum (Figure 6b). Based on the integrated area of those peaks, the amount of pyridinic-N was calculated to reach 62%. It is worth mentioning that no signals corresponding to Zn_3_N_2_ were seen, suggesting that the nitrogen atoms were effectively introduced into the carbon matrix [48]. Similar to the ZIF-8, the spectrum of Zn 2p (see Figure 6c) displays two typical peaks at (1) 1021.7 eV (Zn 2p_3/2_) and (2) 1044.8 eV (Zn 2p_1/2_). Since the difference in binding energy between those peaks was also 23.1 eV, the magnified Zn 2p_3/2_ peak was further fitted to distinguish the chemical bonding among the Zn species. As revealed in Figure 6d, it can be deconvoluted into two peaks: (1) Zn^0^ at 1021.3 eV and (2) ZnO at 1022.0 eV [49]. The integrated area ratio of Zn^0^ to ZnO was calculated to be 1.1, signifying that the Zn that existed within the *N,Z*-MPC was in metallic form due to the thermal reduction [50]. However, it was partially oxidized into ZnO when exposing the *N,Z*-MPC to air. Despite that, these Zn-based sites confining within the *N,Z*-MPC would give an excellent affinity and strong interaction with sodium ions upon cycling [51].

According to the results disclosed in this section, the *N,Z*-MPC acquired after thermal pyrolysis of ZIF-8 under an argon atmosphere gives diverse benefits, including enlarged *d*-spacing, a high proportion of sp^2^-type carbon, distinctive textural feature, a great amount of nitrogen dopant, and sodiophilic Zn species. Encouraged by those characteristics, it would make *N,Z*-MPC a very favorable material for sodium storage applications. What is more is that exploring the sodium storage mechanism within such an *N,Z*-MPC is also of significance.

### 2.3. Electrochemical Measurements of Nitrogen-Doped and Zinc-Confined Microporous Carbon (N,Z-MPC)

**Figure 7:**
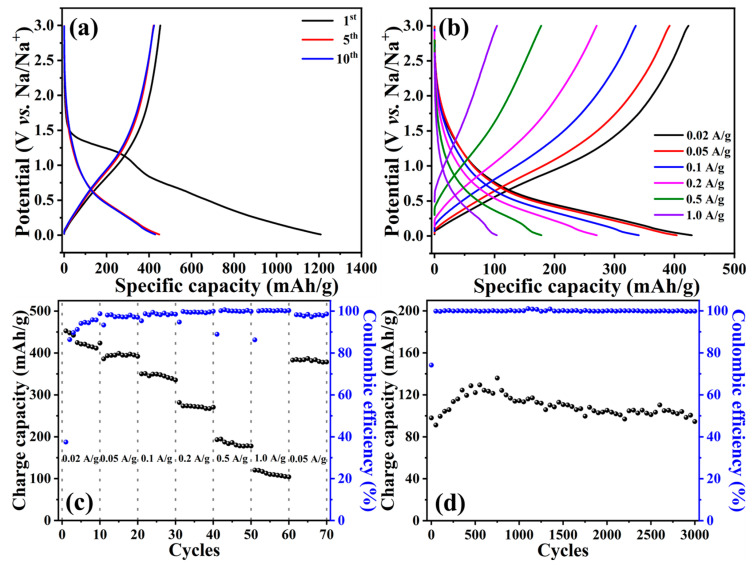
Electrochemical performances of *N,Z*-MPC: (**a**) Galvanostatic charge-discharge profiles collected at 0.02 A/g, (**b**) Galvanostatic charge–discharge profiles recorded at the current densities from 0.02 A/g to 1.0 A/g, (**c**) charge capacities vs. cycles at various current densities, and (**d**) cycling stability measured at 1.0 A/g.

To evaluate the Na^+^-storage performance of *N,Z*-MPC, it was galvanostatically tested in the potential window between 0.01 V and 3.0 V (*vs.* Na/Na^+^) at a current density of 0.02 A/g. As depicted in Figure 7a, the charge and discharge capacities in the first cycle were 453 mAh/g and 1209 mAh/g, corresponding to ~38% of the initial Coulombic efficiency. In addition, not only a distinct plateau appeared around 1.3 V (*vs.* Na/Na^+^) but also a sloping potential ranging from 0.8 V to 0.01 V (*vs.* Na/Na^+^) was observed. Those were also reflected in the CV curve (refer to the first cycle of Figure S7). The former indicates the reaction between sodium ions and active sites, whereas the latter associates with electrolyte decomposition and solid electrolyte interface (SEI) formation [52]. Generally, such an obvious irreversibility in the initial cycle is ascribed to the latter one. In the coming cycles, another sloping potential between 0.6 V and 0.01 V (*vs.* Na/Na^+^) was displayed, which was attributed to the insertion/extraction of sodium ions through the carbon lattices and also in good agreement with the CV results. It is worth mentioning that the average potential for inserting sodium ions into *N,Z*-MPC was higher than that for previously reported carbonaceous materials (between 0 and 0.1 V vs. Na/Na^+^), which would avoid the growth of dendrites [53]. By considering the Na^+^-storage capacity that was contributed above 0.6 V (*vs.* Na/Na^+^), it was about 140 mAh/g at the 10th cycle, i.e., ~33% of the total. This significant enhancement can be acknowledged to the existence of pyridinic-N and pyrrolic-N, evidenced in Figure 6b, increasing the active sites for rapid Na^+^ adsorption.

Figure 7b illustrates the galvanostatic charge-discharge (GCD) profiles measured at current densities from 0.02 A/g to 1.0 A/g. As can be seen, their GCD tendencies were not significantly varied even after gradually increasing the rates. Additionally, the ratio between the discharge capacities delivered at 0.6 V (*vs.* Na/Na^+^) to the total was kept at ~33%, as plotted in Figure S8, inferring the stable and reversible Na^+^-storage kinetic within the *N,Z*-MPC. According to the GCD curves, the charge capacities of 423, 392, 335, 270, 178, and 104 mAh/g at 0.02, 0.05, 0.1, 0.2, 0.5, and 1.0 A/g, respectively, are compared in Figure 7c. When the current density was returned to 0.05 A/g, it was noticed that 96.4% of the recovery in capacity was achieved. Figure S9a compares the EIS spectra that were recorded before and after rate-capability measurements. Before testing, the charge transfer resistance (*R*_ct_) was fitted to be 453 Ohm according to the equivalent circuit model shown in Figure S9b. Following the repeated charge-discharge processes, the resistance that came from the SEI layer (*R*_SEI_) was about 70 Ohm, in which the *R*_ct_ value was also increased to 828 Ohm (see Figure S9c for the fitting model). It is recognized that the *R*_ct_ included ionic and electronic resistances. The former is the resistance to the ionic mobility inside the textual pores of the electrode, while the latter comprises the intrinsic resistance of the electrode material and the contact resistance between the active layer and the current collector [54]. The *N,Z*-MPC still displayed good reversibility even though ~83% of the increase in the *R*_ct_ appeared, which could be supported by its distinctive features, i.e., microporosity, a great amount of nitrogen doping, high proportions of sp^2^-type carbons, etc. The cycling stabilities were further measured at 0.2 and 1.0 A/g, while the corresponding results are shown in Figure S10 and Figure 7d, respectively. As revealed, the initial charge capacity was 272 mAh/g with ~91% of Coulombic efficiency when the *N,Z*-MPC was cycled at 0.2 A/g. Even though the capacity values fluctuated, the capacity retention was calculated to be 92.6% after 300 cycles. Encouragingly, 96.6% of capacity retention was further noticed at 3000 cycles at 1.0 A/g, as depicted in Figure 7d. These electrochemical performances were further compared with previously reported nitrogen-doped carbon anodes using the carbonate-based electrolyte. As summarized in Table S2, it is clearly noticed that the initial Coulombic efficiency, rate capabilities, and capacity retention were significantly influenced by the N content and specific surface area. Of course, other parameters such as the electrode’s packing density, material’s porosity, and morphology also varied the corresponding electrochemical performances. Nevertheless, the *N,Z*-MPC still shows better reversible capacity recorded at 0.05 A/g with other studies [55,56,57,58,59,60].

## 3. Materials and Methods

### 3.1. Chemicals

All reagents, including zinc acetate dihydrate (Zn(CH_3_COO)_2_·2H_2_O, ≥98%, Sigma-Aldrich, St. Louis, MO, USA), 2-methylimidazole (C_4_H_6_N_2_, 99%, Sigma-Aldrich, St. Louis, MO, USA), sodium perchlorate (NaClO_4_, anhydrous, 98%, Alfa Aesar, Heysham, England), ethylene carbonate (EC, 99%, Alfa Aesar, Heysham, England), diethyl carbonate (DEC, 99%, Alfa Aesar, Heysham, England), fluoroethylene carbonate (FEC, 99%, Sigma-Aldrich, St. Louis, MO, USA), poly(vinylidenedifluoride) (PVDF, Kynar^®^ HSV 900, Arkema Inc., Colombes, France), N-methylpyrolidinone (NMP, ChromAR^®^, Macron Fine Chemicals^TM^, Radnor, PA, USA), sodium metal (Na, 99%, E-Current CO., LTD., Taipei, Taiwan), glass fiber separator (Type A/E, P/N 61630, Pall corporation, Port Washington, NY, USA), and carbon black (Super P^®^, Timcal Ltd., Bodio, Switzerland) were employed without further purification. Deionized (DI) water produced from a Milli-Q^®^ Integral water purification system (Millipore Ltd., Burlington, MA, USA) was used throughout the experiments.

### 3.2. Synthesis of Zeolitic Imidazolate Framework-8 (ZIF-8)

To synthesize the ZIF-8, 2 g of Zn(CH_3_COO)_2_·2H_2_O was first dissolved in 30 mL of DI water to give solution A. Subsequently, solution A was directly added into another solution that contained 7.48 g of C_4_H_6_N_2_ and 30 mL of DI water with continuous stirring for 16 h under ambient conditions. The white ZIF-8 powders (~1.8 g) were finally acquired by centrifugation, rinsing with DI water, freeze-drying, and grinding.

### 3.3. Preparation of Nitrogen-Doped and Zinc-Confined Microporous Carbon (N,Z-MPC)

For preparing the *N,Z*-MPC, 1.6 g of ZIF-8 powders were thermally pyrolyzed in the tube furnace at 800 °C for 3 h under an argon atmosphere with a flow rate of 200 mL/min. The black *N,Z*-MPC powders were collected when they were naturally cooled down to room temperature and thoroughly ground using a pestle and agate mortar. The yield of *N,Z*-MPC was about 50%, which was attributed to the thermal decomposition of organic ligands within the ZIF-8.

### 3.4. Characterizations

The crystalline structures of ZIF-8 and *N,Z*-MPC were identified using a powder X-ray diffractometer (PXRD, Bruker D2 PHASER) with a Cu target (λ = 1.541 Å) that was excited at 30 kV and 10 mA. In addition, the corresponding PXRD pattern of ZIF-8 recorded in the range of 2*θ* from 5° to 50° at a scanning rate of 2.5 sec/step was further refined via Rietveld analysis using TOPAS 4.2 software (Bruker AXS Inc., Karlsruhe, Germany). As for the *N,Z*-MPC, the PXRD pattern was collected from 2*θ* between 10° and 70° at a scanning rate of 0.5 s/step. For morphological observations, the scanning electron microscope (SEM, JSM-IT200, JEOL Ltd., Tokyo, Japan) and transmission electron microscope (TEM, JEM-2100, JEOL Ltd., Tokyo, Japan) operated at an accelerating voltage of 200 kV was utilized. N_2_ adsorption–desorption isotherm was recorded at 77 K on a surface area and porosity analyzer (ASAP 2020 V3.00, Micromeritics Instrument Corporation, Norcross, GA, USA) after the ZIF-8 and *N,Z*-MPC had been degassed in a vacuum at 160 °C for 8 h. The pore size distribution of each sample was analyzed using a *t-plot* method (correlation coefficient ≥ 0.995), which is a well-known technique for determining the micro- and/or mesoporous volumes [61]. The chemical environments were analyzed through an X-ray photoelectron spectroscopy (XPS, PHI 5000 VersaProbe III, ULVAC-PHI, Inc., Kanagawa, Japan) with a beam size of 100 um under Al Kα radiation (λ = 8.3406 Å). Their corresponding spectra were analyzed by the Gaussian-Lorentzian fitting method using XPSPEAK 4.1 software. The Raman spectrum was collected by a confocal Raman microscope (inVia, Renishaw, UK) equipped with a 633 nm laser source.

### 3.5. Electrochemical Measurements

The electrochemical tests throughout this study were conducted using the CR2032 coin-type cells under ambient conditions. The working electrodes were prepared by homogeneously blending 70 wt.% *N,Z*-MPC, 10 wt.% Super P, and 20 wt.% PVDF with the proper volume of NMP solvent. The resulting slurry was then coated onto the carbon-coated copper foil using a doctor-blade process and dried at 60°C until the NMP was completely evaporated. The mass loading of *N,Z*-MPC was 0.3 ~ 0.6 mg/cm^2^. Meanwhile, sodium foil, 1 M NaClO_4_/EC:DEC (1:1 v/v) with 5 vol.% FEC, and glass fiber served as the counter electrode, electrolyte, and separator, respectively. After assembling the half cells, the potential range utilized for cyclic voltammetry (CV) and galvanostatic charge-discharge (GCD) measurements was between 0.01 V and 3.0 V (*vs.* Na/Na^+^). The cyclic voltammograms and electrochemical impedance spectroscopy (EIS) spectra were recorded using a multichannel electrochemical workstation (VSP-3e, Bio-Logic, Seyssinet-Pariset, France). The latter was performed at open circuit potential (OCP) from 100 kHz to 0.01 Hz with an AC potential amplitude of 5 mV. The GCD profiles and cycling stabilities were evaluated through a computer-controlled system (CT-4008T-5V50mA, Neware Technology Limited, Shenzhen, China). The capacity values presented throughout this study were calculated based on the total mass of *N,Z*-MPC.

## 4. Conclusions

In summary, the polyhedral ZIF-8 nanoparticles with great crystallinity, high purity, and huge specific surface area were successfully synthesized utilizing DI water as the green medium under ambient conditions. Through thermal pyrolysis of synthesized ZIF-8 under an argon atmosphere at 800 °C, the nitrogen-doped and zinc-confined microporous carbon (*N,Z*-MPC) powders were subsequently obtained to explore the Na^+^-storage performances. Following the systemic characterizations, the *N,Z*-MPC exhibited great dis-ordered structure, enlarged interplanar distance, and a high proportion of sp^2^-type carbon. Combining those characteristics with major pyridinic-N configuration, sodiophilic Zn species, and microporous feature, our *N,Z*-MPC delivered good reversible capacities (335 mAh/g at 0.1 A/g), comparable rate capabilities (104 mAh/g at 1.0 A/g), and remarkable cyclabilities (96.6% of capacity retention at 3000 cycles at 1.0 A/g, i.e., 0.001% of loss per cycle). Consequently, the results shown in this study suggested that the *N,Z*-MPC can thus be prepared as a promising anode material with favorable Na^+^-storage abilities toward sodium-ion batteries/capacitors applications.

## Data Availability

Data sharing is not applicable to this article.

## Supplementary Materials

The following supporting information can be downloaded at: https://www.mdpi.com/article/10.3390/ijms24108777/s1, Figure S1: (a) SEM micrograph and (b) nitrogen adsorption–desorption isotherm of ZIF-8. Scale bar of (a) is 500 nm, whereas the inset of (b) shows the pore-size distribution analyzed by a *t-plot* method. Figure S2: Full-range XPS spectrum of ZIF-8. Figure S3: The magnified XRD pattern of (002) peak for *N,Z*-MPC analyzing by Gaussian-Lorentzian method. Figure S4: (a) SEM micrograph and (b) nitrogen adsorption–desorption isotherm of *N,Z*-MPC. Scale bar of (a) is 500 nm, whereas the inset of (b) shows the pore-size distribution analyzed by a *t-plot* method. Figure S5: SEM-EDX analysis of *N,Z*-MPC, scale bar: 250 nm. Figure S6: Full-range XPS spectrum of *N,Z*-MPC. Figure S7: Cyclic voltammograms of *N,Z*-MPC collected in the potential range between 0.01 V and 3.0 V (*vs.* Na/Na^+^) at a scanning rate of 0.2 mV/sec. Figure S8: Relationship between the discharge capacity contributed from the potential below and above 0.6 V (*vs.* Na/Na^+^) and current density. Figure S9: (a) Electrochemical impedance spectra of *N,Z*-MPC, and the equivalent circuit models used for parameter fitting: (b) before and (c) after C-rate measurements. Figure S10: The cycling stability of *N,Z*-MPC measured at 0.2 A/g. Table S1: Parameters of (002) peak from XRD pattern and related profile-fitting results. Table S2: Comparisons of the electrochemical performances on previously reported nitrogen-doped carbon anodes for sodium-ion batteries using the carbonate-based electrolyte [20,43,55,56,57,58,59,60,62,63].
