# Supplementary material for: In Situ Construction of Nitrogen-Doped and Zinc-Confined Microporous Carbon Enabling Efficient Na+-Storage Abilities
Source: Int J Mol Sci. 2023 May 15;24(10):8777. doi: 10.3390/ijms24108777 (PMC10218166; doi:10.3390/ijms24108777)
Supplement: Supplementary file 1 [file ijms-24-08777-s001.zip › ijms-2393688-supplementary.pdf]

Supplementary Information

Article

In Situ Construction of Nitrogen-Doped and Zinc-Confined Microporous Carbon Enabling Efficient Na^+-Storage Abilities

Wan-Ling Liao [1], Mohamed M. Abdelaal [1,2], Rene Mary Amirtha [1], Chia-Chen Fang [3], Chun-Chen Yang [1,4,5] and Tai-Feng Hung [1,*]

[1] Battery Research Center of Green Energy, Ming Chi University of Technology, 84 Gungjuan Rd., New Taipei City 24301, Taiwan
[2] Tabbin Institute for Metallurgical Studies (TIMS), Tabbin, Helwan 109, Cairo 11421, Egypt
[3] Material and Chemical Research Laboratories, Industrial Technology Research Institute, 195, Sec. 4, Chung Hsing Rd., Hsinchu 31040, Taiwan
[4] Department of Chemical Engineering, Ming Chi University of Technology, 84 Gungjuan Rd., New Taipei City 24301, Taiwan
[5] Department of Chemical and Materials Engineering, Chang Gung University, 259 Wenhua 1st Rd., Taoyuan 33302, Taiwan
* Correspondence: taifeng@mail.mcut.edu.tw; Tel.: +886-2-2908-9899 (ext. 4957)

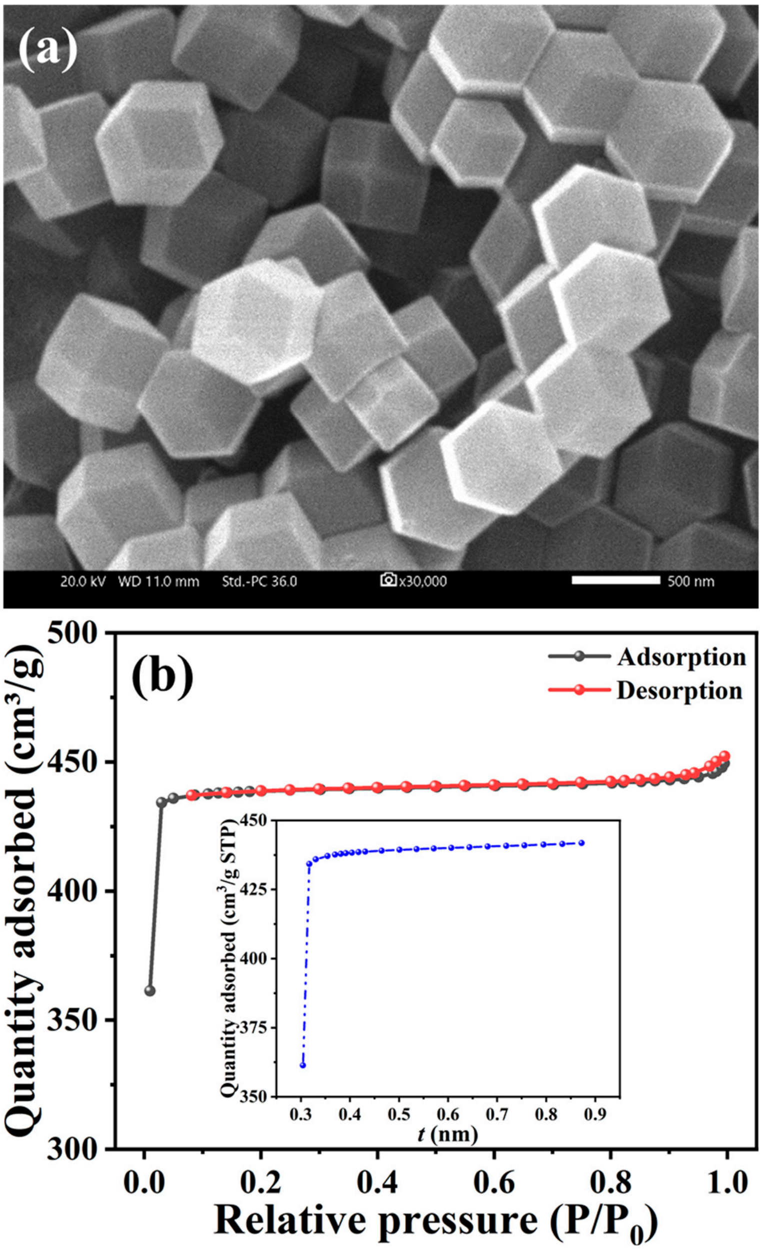

Figure S1. (a) SEM micrograph and (b) nitrogen adsorption-desorption isotherm of ZIF-8. Scale bar of (a) is 500 nm, whereas the inset of (b) shows the pore-size distribution analyzed by a *t-plot* method.

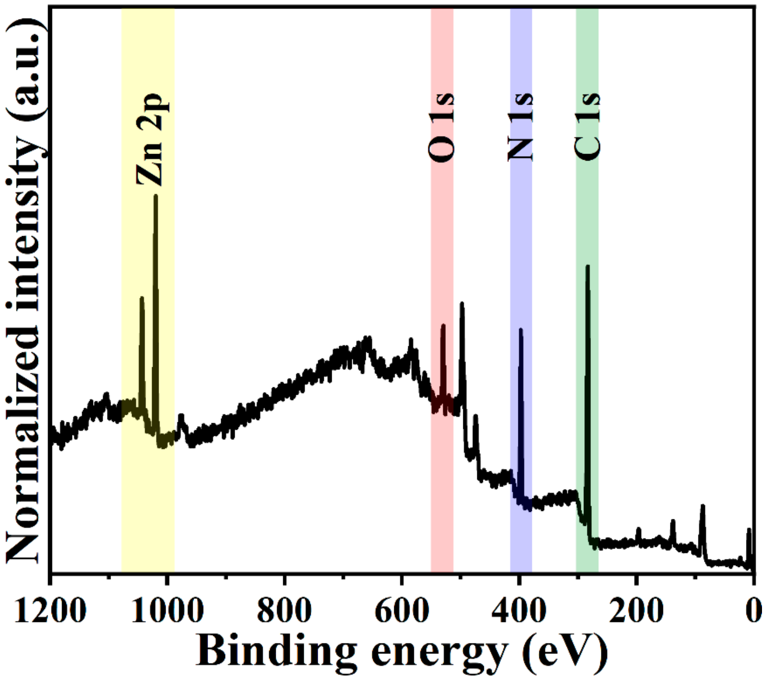

Figure S2. Full-range XPS spectrum of ZIF-8.

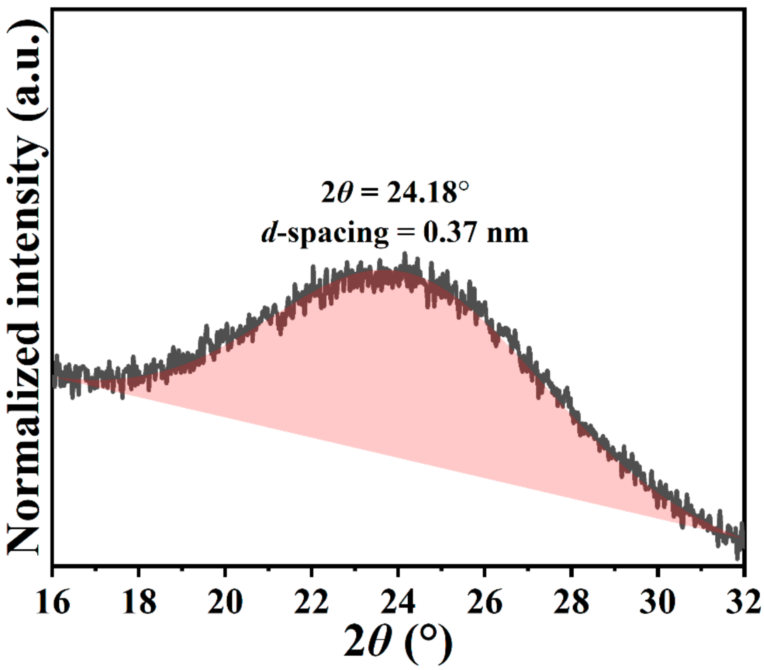

Figure S3. The magnified XRD pattern of (002) peak for *N,Z*-MPC analyzing by Gaussian-Lorentzian method.

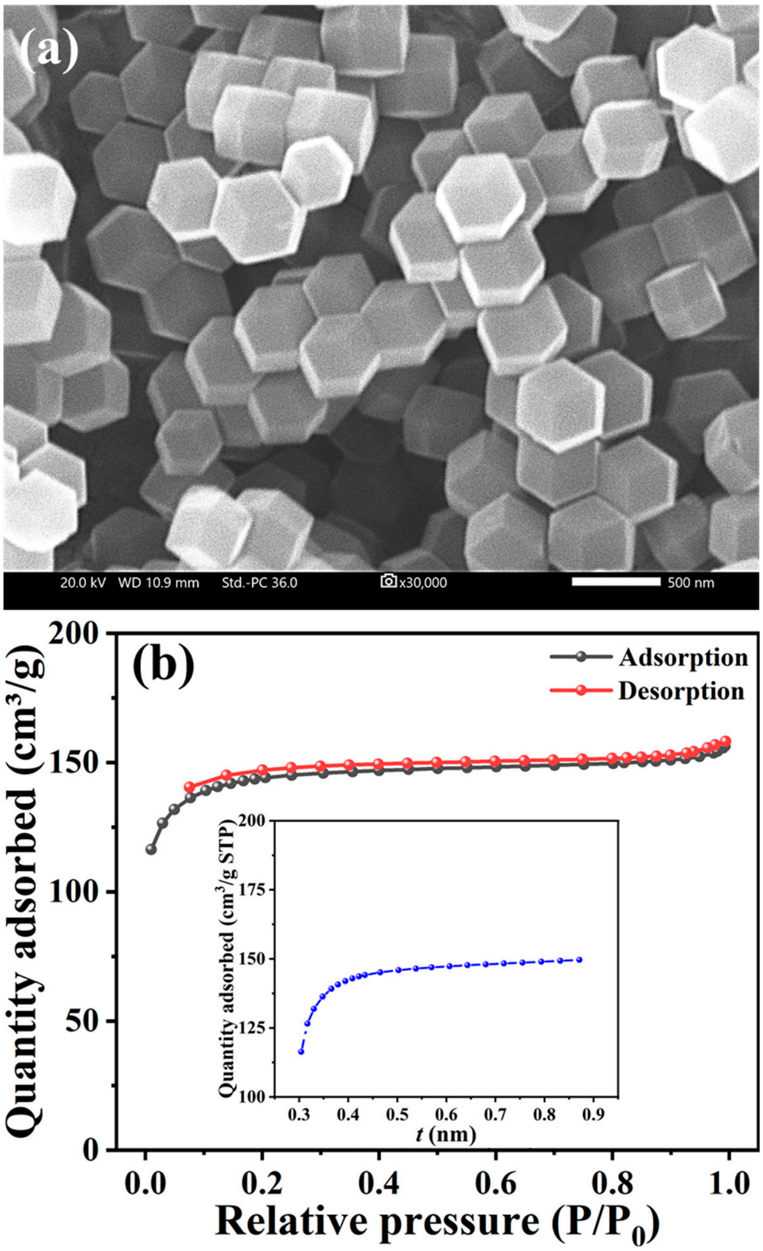

Figure S4. (a) SEM micrograph and (b) nitrogen adsorption-desorption isotherm of *N,Z*-MPC. Scale bar of (a) is 500 nm, whereas the inset of (b) shows the pore-size distribution analyzed by a *t-plot* method.

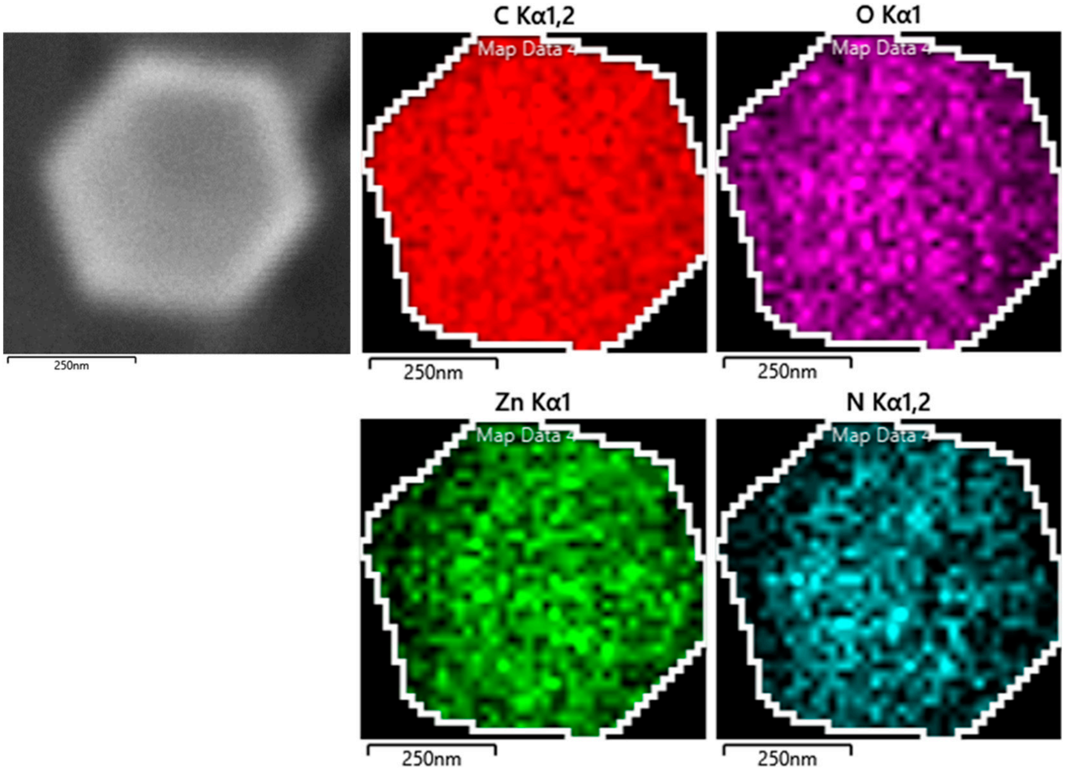

Figure S5. SEM-EDX analysis of *N,Z*-MPC, scale bar: 250 nm.

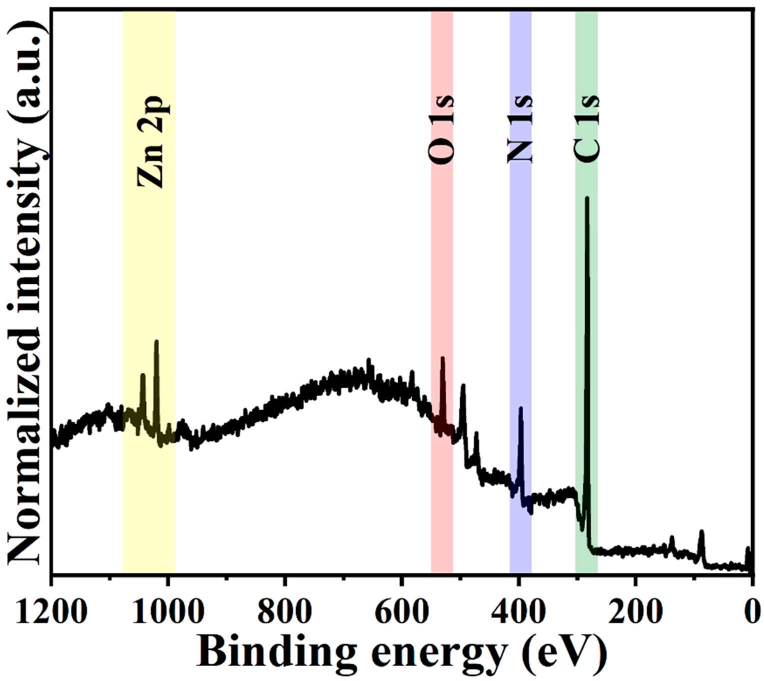

Figure S6. Full-range XPS spectrum of *N,Z*-MPC.

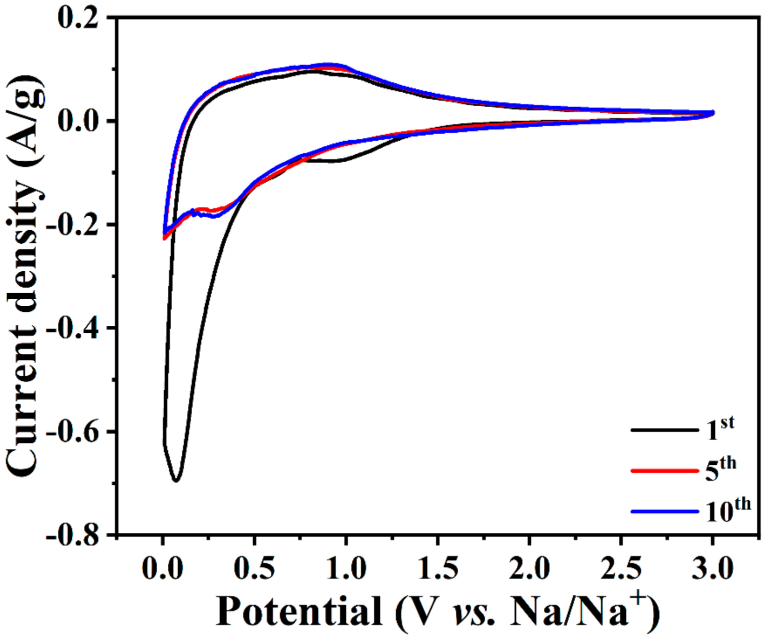

Figure S7. Cyclic voltammograms of *N,Z*-MPC collected in the potential range between 0.01 V and 3.0 V (*vs.* Na/Na^{+}) at a scanning rate of 0.2 mV/sec.

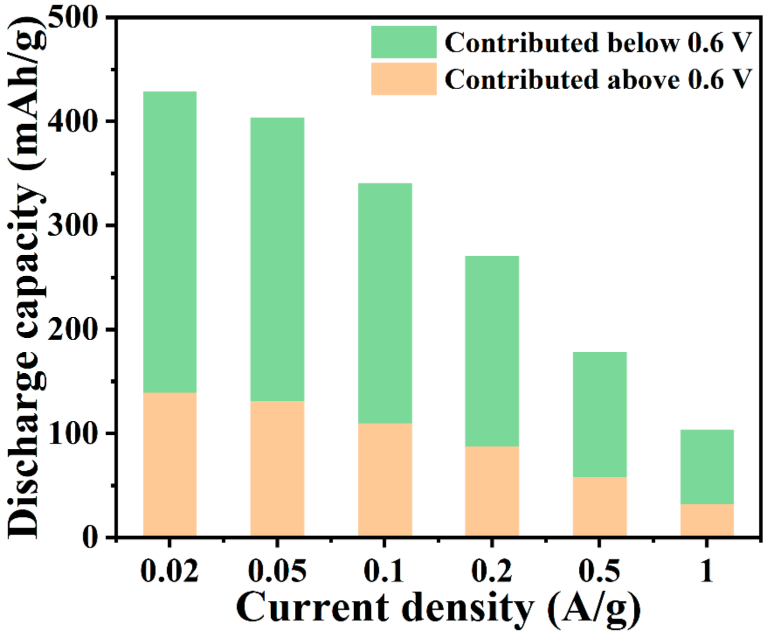

Figure S8. Relationship between the discharge capacity contributed from the potential below and above 0.6 V (*vs.* Na/Na^{+}) and current density.

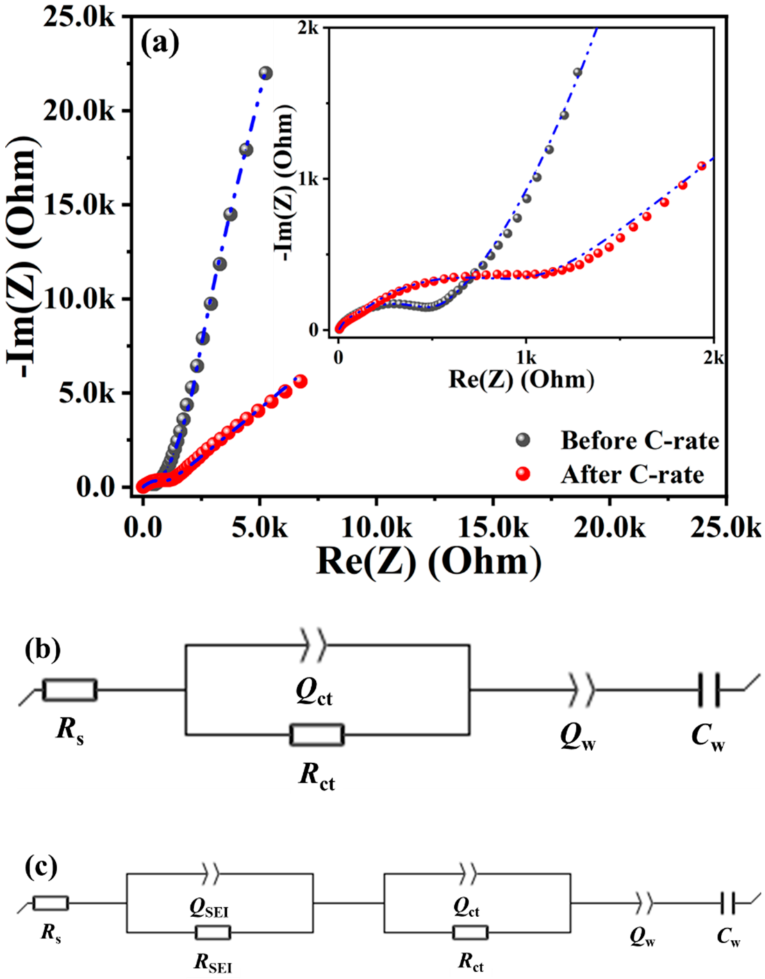

Figure S9. (a) Electrochemical impedance spectra of *N,Z*-MPC, and the equivalent circuit models used for parameter fitting: (b) before and (c) after C-rate measurements.

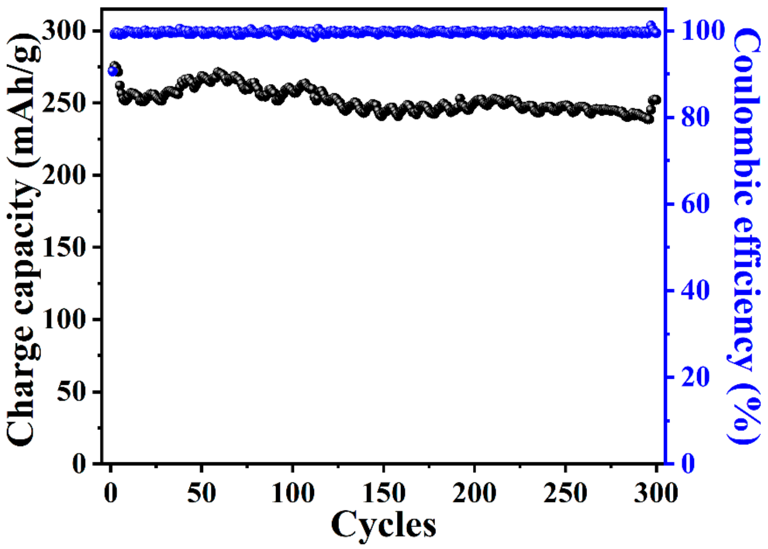

Figure S10. The cycling stability of *N,Z*-MPC measured at 0.2 A/g.

Table S1. Parameters of (002) peak from XRD pattern and related profile-fitting results.

	(002) peak		Graphitic region			Disordered region			
Sample	2θ (º)	d_{002}† (nm)	2θ (º)	d_{002}† (nm)	Area (%)	2θ (º)	d_{002}† (nm)	Area (%)	R‡
N,Z-MPC	24.18	0.37	26.37	0.34	33	23.05	0.39	67	2.96

†Calculated by Bragg equation with the center of (002) diffraction peak.

‡Defined as the ratio of the height of the (002) diffraction peak to the background.

Table S2. Comparisons of the electrochemical performances on previously reported nitrogen-doped carbon anodes for sodium-ion batteries using the carbonate-based electrolyte.

N content† (%)	S_{BET} (m^2/g)	Initial Coulombic efficiency (%)	Specific capacity (mAh/g at A/g)	Capacity retention (%)	Ref.
6.5	8	57	435 at 0.05, 147 at 2.0	74 at 1,200 cycles at 1 A/g	[20]
19.5	476	25	335 at 0.05, 94 at 5.0	95 at 265 cycles at 0.05 A/g	[55]
8.6	751	22	310 at 0.05, 54 at 5.0	90 at 2,000 cycles at 1 A/g‡	[56]
N/A	3	N/A	210 at 0.05, 135 at 5.0	71 at 600 cycles at 0.2 A/g	[57]
9	265	66	270 at 0.05, 185 at 1.0	88 at 300 cycles at 1 A/g	[58]
4.6	6	N/A	373 at 0.025, 120 at 1.0	95 at 1,000 cycles at 0.2 A/g	[62]
19.3	82	N/A	264 at 0.1, 57 at 40	99 at 2,000 cycles at 0.5 A/g	[43]
3.9	202	56	332 at 0.1, 135 at 1.6	87 at 300 cycles at 0.2 A/g	[63]
5.8	111	76	285 at 0.05, 153 at 5.0	94 at 600 cycles at 1 A/g	[59]
10.5	212	N/A	263 at 0.05, 92 at 10.0	84 at 2,000 cycles at 0.5 A/g⁋	[60]
16.1	441	38	423 at 0.02, 392 at 0.05, 104 at 1.0	93 at 300 cycles at 0.2 A/g<br>97 at 3,000 cycles at 1.0 A/g	This study

†Determined by XPS, ‡Calculated based on 100th cycle, ⁋Calculated based on 10th cycle.

References:

20. Feng, X.; Bai, Y.; Zheng, L.; Liu, M.; Li, Y.; Zhao, R.; Li, Y.; Wu, C. Effect of different nitrogen configurations on sodium storage properties of carbon anodes for sodium ion batteries. *ACS Appl. Mater. Interfaces* 2021, *13*, 56285-56295.

43. Liu, J.; Zhang, Y.; Zhang, L.; Xie, F.; Vasileff, A.; Qiao, S.-Z. Graphitic carbon nitride (g-C_3N_4)-derived N-rich graphene with tuneable interlayer distance as a high-rate anode for sodium-ion batteries. *Adv. Mater.* 2019, *31*, 1901261.

55. Zhong, X.; Li, Y.; Zhang, L.; Tang, J.; Li, X.; Liu, C.; Shao, M.; Lu, Z.; Pan, H.; Xu, B. High-performance sodium-ion batteries based on nitrogen-doped mesoporous carbon spheres with ultrathin nanosheets. *ACS Appl. Mater. Interfaces* 2019, *11*, 2970-2977.

56. Qu, Y.; Guo, M.; Wang, X.; Yuan, C. Novel nitrogen-doped ordered mesoporous carbon as high-performance anode material for sodium-ion batteries. *J. Alloys Compd.* 2019, *791*, 874-882.

57. Xue, K.; Si, Y.; Xie, S.; Yang, J.; Mo, Y.; Long, B.; Wei, W.; Cao, P.; Wei, H.; Guan, H.; et al. Free-standing N-doped porous carbon fiber membrane derived from Zn–MOF-74: Synthesis and application as anode for sodium-ion battery with an excellent performance. *Front. Chem.* 2021, *9*.

58. Yanilmaz, M.; Atıcı, B.; Zhu, J.; Toprakci, O.; Kim, J. N-doped carbon nanoparticles on highly porous carbon nanofiber electrodes for sodium ion batteries. *RSC Adv.* 2023, *13*, 7834-7842.

59. Shan, C.; Feng, X.; Yang, J.; Yang, X.; Guan, H.-Y.; Argueta, M.; Wu, X.-L.; Liu, D.-S.; Austin, D.J.; Nie, P.; et al. Hierarchical porous carbon pellicles: Electrospinning synthesis and applications as anodes for sodium-ion batteries with an outstanding performance. *Carbon* 2020, *157*, 308-315.

60. Zhang, J.; Zhang, Q.; Qu, X.; Xu, G.; Fan, B.; Yan, Z.; Gui, F.; Yang, L. Hierarchically pyridinic-nitrogen enriched porous carbon for advanced sodium-ion and lithium-sulfur batteries: Electrochemical performance and in situ Raman spectroscopy investigations. *Appl. Surf. Sci.* 2022, *574*, 151559.

62. Nie, W.; Cheng, H.; Liu, X.; Sun, Q.; Tian, F.; Yao, W.; Liang, S.; Lu, X.; Zhou, J. Surface organic nitrogen-doping disordered biomass carbon materials with superior cycle stability in the sodium-ion batteries. *J. Power Sources* 2022, *522*, 230994.

63. Chen, Y.; Wu, Y.; Liao, Y.; Zhang, Z.; Luo, S.; Li, L.; Wu, Y.; Qing, Y. Tuning carbonized wood fiber via sacrificial template-assisted hydrothermal synthesis for high-performance lithium/sodium-ion batteries. *J. Power Sources* 2022, *546*, 231993.
